# Fabrication of a Malaria-Ab ELISA Bioassay Platform with Utilization of Syringe-Based and 3D Printed Assay Automation

**DOI:** 10.3390/mi9100502

**Published:** 2018-10-02

**Authors:** Christopher Lim, Yangchung Lee, Lawrence Kulinsky

**Affiliations:** 1 Department of Chemical Engineering and Materials Science, University of California, Irvine, 916 Engineering Tower, Irvine, CA 92627-2575, USA; cwplim@gmail.com (C.L.); Yangchung_Lee@amat.com (Y.L.); 2 Department of Mechanical and Aerospace Engineering, University of California, Irvine, 5200 Engineering Hall, Irvine, CA 92627-2700, USA

**Keywords:** diagnostics, liquid handling, microfluidics, multiplex assays and technology, stereolithography, sample preparation, 3D printing

## Abstract

We report on the fabrication of a syringe-based platform for automation of a colorimetric malaria-Ab assay. We assembled this platform from inexpensive disposable plastic syringes, plastic tubing, easily-obtainable servomotors, and an Arduino microcontroller chip, which allowed for system automation. The automated system can also be fabricated using stereolithography (SLA) to print elastomeric reservoirs (used instead of syringes), while platform framework, including rack and gears, can be printed with fused deposition modeling (FDM). We report on the optimization of FDM and SLA print parameters, as well as post-production processes. A malaria-Ab colorimetric test was successfully run on the automated platform, with most of the assay reagents dispensed from syringes. Wash solution was dispensed from an SLA-printed elastomeric reservoir to demonstrate the feasibility of both syringe and elastomeric reservoir-based approaches. We tested the platform using a commercially available malaria-Ab colorimetric assay originally designed for spectroscopic plate readers. Unaided visual inspection of the assay solution color change was sufficient for qualitative detection of positive and negative samples. A smart phone application can also be used for quantitative measurement of the assay color change.

## 1. Introduction

### 1.1. Preface

Lab-on-a-chip systems are used to perform personalized health diagnostic tests (like bioassays) away from the lab [1]. Implementation of bioassay automation reduces the cost of medical personnel and diminishes the incidence of human errors [2,3]. Point-of-care (POC) diagnostic platforms are required to have low cost, low power use, be reliably automated, and free of sophisticated detection technologies [4]. POC platforms have already been realized in the form of lateral flow immunoassays (pregnancy tests) and paper fluidic colorimetric assays [5]. Unfortunately, the aforementioned POC devices are based on capillary flow, and therefore do not work well when more complex multi-step bioassays are performed [6]. In these cases, some other fluid propulsion mechanisms instead of capillary flow are required. There are many POC devices that rely on active propulsion techniques, like centrifugal propulsion, electrowetting, and magnetic beads [7,8]. These tests typically entail increased fabrication and material cost, complex automation schemes, and sophisticated hardware.

In this work, we developed two distinct approaches for the realization of an inexpensive automated colorimetric immunoassay with multiple wash steps: (1) A fused deposition modeling (FDM)-printed (non-disposable) frame was used and a disposable fluidic chip that includes an elastomeric dome and fluidic channels fabricated using SLA, was printed, where the fluidic movement was facilitated by servomotors pushing on the elastomeric dome, and propelling the reagents or wash from the domed reservoirs through fluidic channels to the test chamber, or (2) an FDM-printed non-disposable frame was used, with servomotors connected to standard inexpensive and readily-available disposable plastic syringes filled with wash and reagents to automate the steps of the assay.

Draining of the test chamber was performed with a syringe attached to a servomotor, where negative pressure was created by the pulling on the syringe plunger. All automation was controlled by a program uploaded to the Arduino-based electronic board of the platform via a computer. The test results of the colorimetric bioassay can be assessed by eye (for qualitative measurements) or with a smart phone for quantitative measurement, and e-mailed or texted to a hospital or doctor’s office [9,10]. In this work, we created a proof-of-principle platform that utilized elements of both approaches (1) and (2), as outlined above.

We used plastic syringes for most of the platform reagent reservoirs, including drainage and waste. The wash reservoir consisted of an SLA-printed elastomeric dome. Thus, the feasibility of both approaches was tested at the same time. The automated platform demonstrated in the presented work proved the viability of both approaches: (1) Construction of an automated bioassay platform using syringes only, or (2) a printed dome-based bioassay platform. To our knowledge, this study constitutes the first demonstration of an SLA-based dome bioassay platform and a syringe-based automated bioassay platform. The concepts developed in the present work build on prior research that utilized FDM to fabricate a fluidic chip with embedded microchannels [11]. Fluid leakage was a recurring issue in the design, due to the layer-by-layer deposition nature of FDM. Our present fabrication approach utilizes syringes and photocurable resin crosslinked using SLA, thus mitigating the problem of fluid leakage. The present platform employs FDM printing for the fabrication of parts that are not in contact with fluids, such as the non-disposal platform frame and the gear/rack mechanisms. Additionally, the prior fluidic platform design used molded silicone domes that needed to be sealed to the body of 3D printed fluidic chip. Our present fabrication route avoids the need to seal the domes to the plastic chip by 3D-printing the integrated dome.

The proposed approach is useful in making POC bioassays more widespread. All of the parts and materials required for the demonstrated platforms are inexpensive and widely available. For example, a hospital in rural India with an FDM printer could be sent a stereolithography file (STL file) to print a frame. The Arduino electronic board and other parts, such as servomotors, can be easily acquired. All the parts and materials to produce our automated platform cost less than $100, while all disposable materials, including syringes, tubing, and reagents, cost around $5 per test.

If an exclusively syringe-based platform is utilized, the only disposable parts needed are plastic syringes. The syringes would be filled with a prescribed volume of reagents, and the required program uploaded to the Arduino board. This approach provides the most flexibility to perform a wide range of bioassays on the spot, as it does not require all the reagents in typical pre-packaged volumes. This simplifies assay logistics, including that of cold storage of reagents [12]. We expect that our proposed recipes and programs will be readily available for download by non-profit organizations and reagent makers.

There is a growing interest in manufacturing functional parts inexpensively with high-aspect-ratio geometry and complex topologies [13]. Additive manufacturing (AM), including various types of 3D printing, is an increasingly popular form of fabrication [14]. The AM process requires a part to be designed using a CAD program, such as SolidWorks, followed by the extrapolation of the structural information into an STL file. The STL file partitions the CAD drawing into a series of volumetric pixels, which are digitally sliced into layers along the Z direction [15]. These layers are physically deposited onto a substrate in layer-by-layer manner, using various AM methods, including FDM and SLA. FDM and SLA accommodate a variety of material choices, including elastomeric materials, allowing for the fabrication of flexible parts such as the dome-based elastic pumps presented in this work.

In this work, we conducted a brief review of microfluidic device fabrication methods, and emphasized recent developments in additive manufacturing. We followed this review with a description of the fabrication processes selected for our bioassay platform. Lastly, we detailed an outline of our experimental procedures, and described the experimental results of a malaria bioassay performed on our automated platform.

### 1.2. Fabrication Techniques for Microfluidic Devices

The first traditional microfluidic devices were fabricated using glass and silicon, utilizing mostly a toolbox of lithographic techniques employed in the semiconductor industry [16]. Equipment selection was subsequently further developed to include femtosecond lasers to fabricate glass microfluidic chips [17,18], and the selection of materials employed for fluidic chips was also expanded to include such materials as “liquid Teflon” [19] and environmentally-sensitive materials such as hydrogels [20]. With the advent of soft lithography and 3D printing, there was a push to develop inexpensive and reliable microfluidic platforms with biocompatible plastics and resins, demonstrated by the recent fabrication trends outlined below [21,22,23,24].

#### 1.2.1. Soft Lithography

Soft lithography is based on a three-step process where microfabrication techniques are first used to produce a reusable mold, which is then filled with a curable epoxy Polydimethylsiloxane (PDMS) and subsequently separated from the mold after an appropriate curing time. The molded part is typically attached to a glass substrate using plasma treatment [25]. The soft lithography approach allows for the usage of more expensive high-precision cleanroom lithographic techniques to produce a mold. Once the mold is produced, it can be used multiple times to fabricate identical fluidic chips, dramatically reducing the cost of individual fluidic microdevices.

The soft lithography approach has allowed for much more widespread adoption of lab-on-a-chip devices, and for the production of intricate parts such as elastomeric valves. For example, the PDMS valves used by the Quake group have facilitated the implementation of large-scale microfluidic bioreactors for drug discovery and other applications [26]. However, when the device is assembled from separately produced parts, the manufacturability is restricted, due to cumbersome alignment, bonding, and assembly [24,27].

3D printing presents an approach where a complete microfluidic device is produced without post-processing alignment and assembly steps. Commercial 3D printers are beginning to challenge the resolutions achievable by soft lithography [28].

#### 1.2.2. Inkjet 3D Printing (i3DP)

Inkjet 3D printing is based on inkjet technology and can operate in continuous or drop-on-demand mode. To achieve high accuracy performance in i3DP, there are four critical elements that must be considered: ink material, substrate properties, printing platform, and droplet generation [29]. Inconsistency or change in any of the parameters affects the process reliability. To maintain steady performance, i3DP needs to be used regularly; the clogging of apertures in a print head due to drying of the ink is a persistent problem. There is considerable cost involved in changing from one material to another, since the previous material must be flushed out by the new resin.

One of the greatest advantages of i3DP is its ability to deliver multiple materials at the same time during the fabrication process, allowing for a wide range of material properties (hard and soft plastics, elastomers), and the utilization of inks of different color [30,31]. Creating prototypes with smooth finishes and complex shapes is possible with i3DP; flow channels have been integrated with porous semi-permeable membranes supporting cell culture to study the transport and profiling of drugs [32,33].

Hwang et al. utilized i3DP to print pillars with a diameter of 250 µm and found that the resolution of the process depended on the droplet size, the printer nozzle spacing, and the reflow of the material prior to UV curing. These factors affected the droplet spreading, changing the final dimensions of the printed devices [34].

Paydar et al. examined multi-material 3D printing for microfluidic interconnects. They fabricated a specialized interconnector part, composed of two rigid clamps for the mechanical attachment of a flexible elastomeric O-ring gasket to a microfluidic device. The parts were printed in a single step, eliminating the need for adhesives or additional assembly. While the cost of manufacturing was low, the interconnector was characterized by low maximum sealing pressure due to material fatigue [35].

#### 1.2.3. Two-Photon Polymerization (2PP)

Two-photon polymerization (2PP) is a laser-based technique that utilizes a femtosecond laser to create 3D structures in the bulk of photocurable epoxy resin, employing a highly localized process where two photons are absorbed simultaneously by the molecule being cured [36]. The synergistic effects of optical, chemical, and material non-linearity make it possible to achieve reproducible resolution of tens of nanometers [37].

2PP has shown great potential for fabrication in microfluidics. Kumi et al. described the fabrication of a master for casting PDMS with rectangular microchannels of high aspect ratios by modifying SU-8 resist (material for the master) with a photoacid generator that then allows for the use of 2PP on SU-8 resist. By using the modified resin, the fabrication speed was also increased from 200 µm·s^−1^ to 10,000 µm·s^−1^ with a print time of 1 h. This technique required extensive resin preparation and had a slow build speed [38].

Kawata et al. achieved resolutions of 120 nm, and other attempts included the use of new photo-initiators, a continuous scanning mode, a shorter wavelength, a longer exposure time, and confining the polymerization phenomenon using a quencher molecule [39]. Sugioka et al. conducted a comprehensive review of the fundamentals and fabrication of 3D micro- and nano-components based on 2PP [40].

While 2PP currently produces the highest resolution for microfabricated three-dimensional structures, it is a very time-consuming process: the time required to fabricate a 1 mm^3^ volume microfluidic structure exceeds 104 days [41]. The high cost of femtosecond lasers, positioning systems, optics, and the difficulty of working with multi-material systems are some factors hindering the utilization of 2PP for the mass-production of microfluidic devices.

#### 1.2.4. Fused Deposition Modeling (FDM)

FDM is one of the most widely used additive manufacturing techniques. Many polymers used with FDM techniques are inherently biocompatible [42]. In FDM, filament material is extruded through nozzles and deposited onto a heated substrate [43]. Due to the inherent propensity of melted fiber to solidify as a line, there are limitations in the dimensional accuracy and the surface texture of the produced parts, resulting in a staircase pattern that is ubiquitous on many parts that are produced with FDM.

Lee et al. printed microfluidic features via FDM and evaluated the printing resolution, accuracy, biocompatibility, and surface roughness of acrylonitrile butadiene styrene (ABS) with P430 filament. They found that the accuracy of the printed features had an average deviation of 60.8 µm and 71.5 µm along the Y and X axis, respectively. The surface of the printed channels was rough with protruding filament strands [44].

Kitson et al. fabricated polypropylene reactionware with cylindrical channels 0.8 mm in diameter. The devices could be fabricated in a few hours and could avoid blockages due to the formation of precipitates. The potential of 3D manufacturing was demonstrated by stopping mid-point in the fabrication process to deposit solid reagents into a chamber, which was then sealed with the printer. This is a valuable feature not so easily realized with i3DP or stereolithography [45].

Bishop et al. created a semi-transparent fluidic device using poly(ethyleneterephthalate) with threaded ports, enabling the integration of commercial tubing as well as specially designed 3D fittings [46]. The transparent device included 800 µm × 800 µm square channels. A low-cost desktop Makerbot (New York, NY, USA) 3D printer was used for fabrication of the device. Prussian blue nanoparticles were synthesized in their lab and mixed in a 3D printed channel and applied to electrode surfaces for sensing of H_2_O_2_.

Recently, Dolomite (Royston, UK) launched a production line that offered an FDM printer specifically designed for fabrication of microfluidic platforms [47]. The printer head design allowed for reliable printing of cyclic olefin copolymer (COC). Their new software guided the production to guarantee smooth surface finish inside the channels. This is contrasted with conventional 3D printing, which emphasizes outside surface texture.

#### 1.2.5. Stereolithography (SLA)

SLA is an attractive option for microfluidics due to increasing availability of SLA printers utilizing inexpensive micromirror-based projectors, including some printer kits costing as little as $100 [48]. The performance of SLA printers is quantified by the dimensional accuracy and the surface roughness of the printed object [49,50]. These factors are influenced by the fabrication settings: object orientation, layer thickness, resin properties, and build style. The minimum cross-sectional area of a microchannel made by SLA depends on the laser spot size and the resin viscosity. This resin must be drained post-print [51].

Recent developments have expanded the SLA material selection for a single print to include elastomers and ceramics, while some printers are capable of using multiple resins. It is predicted that in the future it will be possible to use SLA to fabricate metallic sensors and actuators on flexible membranes [51,52,53].

Comina et al. used a Miicraft (Hsinchu, Taiwan) 3D printer to fabricate a reusable mold for PDMS casting [54]. The molds had structures of multiple feature sizes ranging from 50 µm to several mm. The resin had to be manually coated with protective ink to be properly used with PDMS. The Miicraft was also used to print a device with open fluidic channels that are subsequently sealed on top with the adhesive tape. In another study, Comina et al. printed a unibody lab-on-a-chip (LOC) consisting of a separate microfluidic level, and a layer with optical components. The integrated finger pump was used to initiate the preparatory sequence of mixing two reagents and three analytes. The colorimetric glucose sensing assay was read by a smart phone [55].

Wang et al. demonstrated an effective approach to fabricating structural devices using 3D printing. A monomer initiator was added to the Miicraft resin to allow for modification of the surface properties such as hydrophobicity and hydrophilicity [56].

Shallan et al. used a Miicraft printer for the fabrication of a transparent microfluidic device with enclosed 250 µm diameter channels [57]. The dimension of the printed channels had a deviation of 50 to 100 µm from the designed dimensions, and the roof of the sealing channel was rough. This could be improved through changing the curing depth, intensity, exposure wavelength, and time. They argued that inexpensive Miicraft provides a sufficient resolution for most microfluidic devices.

Patrick et al. printed fluidic open channels using a laser-rastering SLA printer (Form1+, Formlabs, Somerville, MA, USA) [58]. They found that the smallest achievable diameter of a circular channel was 900 µm, and the smallest channel side for a square channel was 650 µm. The surface topology was inspected using SEM and visible striations were discovered. Overall, the cost of the materials for each fluidic chip was around $6.

A sample library of standardized microfluidic components was manufactured using SLA by Lee et al. and Bhargava et al. [59,60]. These parts were then used to create a number of modular and reconfigurable microfluidic units. This approach allowed for the creation of complex microfluidic designs based on simple interlocking fluidic modules. The library allowed for the further miniaturization of microfluidic elements and materials.

The Folch lab has printed diaphragm valves and a peristaltic pump integrated within a LOC device printed using SLA with biocompatible Somos WaterShed XC 11122 resin (Elgin, IL, USA) [61]. The valves were leakage free at a closing pressure of 6 psi (provided by the compressed air), and they were operated over many cycles. These valves are regarded as functional modules: two valves can be paired to build a switch, or three valves can be put together in a series to build a pump [62]. However, the portability of these microfluidic devices was limited by the need to use peripherals such as gas canisters.

### 1.3. Combining Several Fabrication Techniques to Manufacture an Automated Fluidic Malaria Enzyme-Linked Immunosorbent Assay (ELISA) Bioassay Platform

Each of the techniques discussed thus far has its own set of advantages and disadvantages. For example, stereolithography, while allowing for higher resolution, is more expensive and less accessible than fused deposition modelling [63]. Therefore, in this work, we applied a combination of fabrication techniques.

In one approach when only an FDM printer is available, we developed a system based on disposable plastic syringes coupled to programmable servomotors. FDM was used to print the non-disposable frame for an automated colorimetric malaria detection test based on enzyme-linked immunosorbent assay (ELISA). In another approach, when the end user has access to an SLA system, we developed a fabrication approach for SLA to produce elastomeric domes with integrated microfluidic channels. Flow of reagents was facilitated by servomotors compressing the elastomeric dome, and propelling the reagents through a microfluidic channel into a connected test chamber. In this approach, the frame was still printed using an FDM printer. We avoided fabricating microfluidic channels using FDM, because filament-based deposition often resulted in a “staircase effect” as discussed previously, causing fabricated structures to be prone to leakages.

## 2. Materials and Methods

### 2.1. Fused Deposition Modeling and Stereolithography

The FDM parts were manufactured using a acrylonitrile butadiene styrene (ABS) filament on a dual extruder AirWolf HD2x printer (Costa Mesa, CA, USA). The printing parameters, including extrusion, travel speeds, and printer head temperature, were optimized as discussed in the “Results and Discussion” section below. A Formlabs’ (Somerville, MA, USA) Form 1+ SLA printer was used to produce the elastomeric domes and integrated fluidic microchannel from proprietary flexible clear resin GPCL02 sold by Formlabs.

### 2.2. Automation of Malaria-Ab Colorimetric ELISA

A malaria-Ab ELISA colorimetric detection kit (IBL International GmbH, Hamburg, Germany) was used to test functionality of the fabricated automated platform. The colorimetric assay was designed to detect antibodies in subjects infected with four *Plasmodium* species that cause malaria. The most significant parasitic diseases in humans are: *P. falciparum*, *P. vivax*, *P. ovale*, and *P. malariae* [64].

The ELISA kit contained clear polystyrene wells coated with recombinant antigens. When the test sample (either serum or plasma) was added to the well, the specific antibodies in the sample combined with antigens in the well. Subsequently, a conjugate solution of recombinant antigens conjugated to horseradish peroxidase was added to the well, and these antigens reacted with the specific antibodies, if they were present.

When the substrate solution of urea peroxide and tetramethyl benzidine was added to the well, it led to a change in solution color from colorless (in case of no specific antibodies present) to blue (when antibodies were present), and finally to yellow when the stop solution was added. It is possible to determine the concentration of the antibodies in the sample by measuring the color intensity with spectral analysis, but in this proof-of-concept study, we limited our experiments to the positive and negative controls provided with the kit. The full details on the recommended volumes of reagents and incubation temperatures were provided by the kit manufacturers and they are summarized in Table 1 [65].

**Figure 1:**
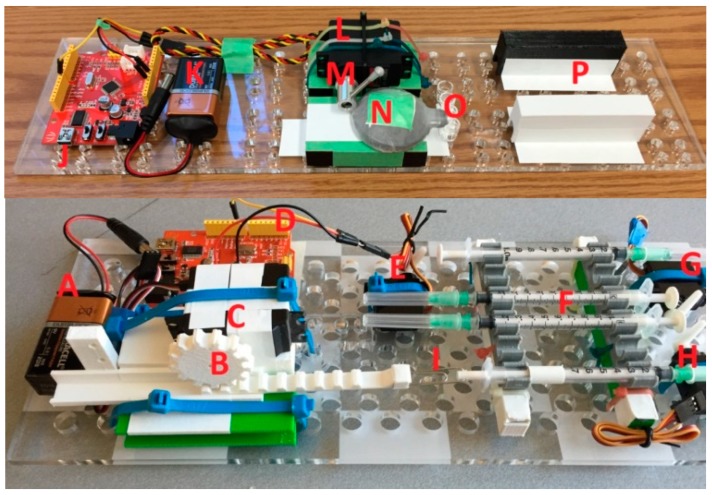
The automated bioassay platform is composed of two stackable boards: The bottom platform contains a battery (A), an aspiration syringe (I) connected via a printed rack-and-gear set (B) to the servomotor (C), which is controlled with an Arduino board (D) that also controls other servomotors (E, G, H) that push on syringes (F) with conjugate, substrate, and stop solutions. The syringes on the bottom board are connected via plastic tubes (4 mm outer diameter, 2 mm inner diameter) to the well (O) on the top platform, where another battery (K) and Arduino board (J) power and control the servomotor (L) that through arm (M), presses on an elastomeric dome (N) containing wash. The top platform also contains a printed frame (P) onto which a smart phone can be placed to detect color changes in the well.

We performed incubation not at the recommended 37 °C, but at a room temperature, usually 20°C to 23 °C. Where the kit instructions recommended draining the test tube and tapping it to make sure that all fluid exited the tube, we implemented automation where a plastic tube is placed within the test well to reach the bottom. This tube was connected to the aspiration syringe (labeled “I” on Figure 1) whose plunger was pulled out by the gear and rack hardware controlled by the servomotor. Pulling of the plunger created the suction necessary to aspirate solutions from the test well.

Each fluidic step of the assay was performed by an action of a servomotor controlled through the Arduino board. The servomotors’ plastic arms pushed onto the plungers of plastic syringes filled with reagents (conjugate solution, substrate, stop solution). We elected to dispense the wash solution from an SLA-printed elastomeric dome containing integrated microfluidic channels, in order to demonstrate an alternative part to syringes for all steps of the assay. The elastomeric dome is easily substituted with another syringe to transition to a completely syringe-based automated fluidic ELISA platform. The complete automated bioassay platform is presented in Figure 1.

The sequence of the bioassay steps is summarized below. Steps of the malaria-Ab ELISA assay as recommended by the kit manufacturer, and the modified steps are executed by the automated platform are listed in Table 1.

(1) Pipette 50 µL of sample into the wells (O). Incubate for 1 hr at room temperature.
(2) Aspirate the sample from the well. Flush the well with wash solution contained within the elastomeric dome (N). The wash solution is dispensed using the movable arm (M) to compress the dome.
(3) Aspirate the dirty wash solution from the well using an aspiration syringe located on the bottom platform. Repeat three times.
(4) Dispense 50 µL of malaria conjugate into the reagent well (O) from a syringe (F). The barrel of this syringe is pushed using a servomotor (H). Incubate for 30 min at room temperature.
(5) Aspirate the conjugate from the well. Repeat step 3.
(6) Dispense 50 µL of the TMB substrate solution into the reagent well (O) from a syringe on the syringe platform (F). The barrel of this syringe is pushed using a servomotor (G). Incubate for 15 min at room temperature.
(7) Dispense 50 µL of stop solution into the reagent well (O) from a syringe on the syringe platform (F). The barrel of this syringe is pushed using a servomotor (E).
(8) Visually inspect the color change if the platform is used for qualitative, rather than quantitative analysis. Alternatively, position a cell phone on the frame (P) and align the phone camera to the reagent wells (O) to read the results using a special colorimetric detection app, Color Catcher (Cloud Innovation Team, Austin, TX, USA) [66]. This app reads RBG values from scanned or photographed images.

### 2.3. Hardware

There were five servomotors used with this automated assay platform: three TowerPro-SG5010 (Shenzhen Hao Qi Core Technology Co., Ltd., Shenzhen, China) servomotors (E, G, and H on Figure 1). These servomotors were used to push onto the plungers of syringes with the stop solution, with TMB substrate, and with the malaria conjugate. A continuous Airtronics Servo 94102 (Sanwa Denshi Co., Tokyo, Japan) (part C in Figure 1) was used in conjunction with a 3D printed rack-and-gear set (B) to pull out the plunger of the aspiration syringe. An Ultra Torque HS-645MG (Hitec RCD USA Inc., Poway, CA, USA) (M) servomotor was used to rotate the arm that pushed on the elastomeric dome (N) containing the wash. The servomotors were programmed to follow the ELISA steps, dispensing the appropriate amount of reagent between each step. These directions were uploaded to the Arduino boards (D, J) (Seeedstudio, Shenzen, China) controlling the servomotors. The arm position of a servomotor was determined by the pulse width-modulated (PWM) signal sent via the control board.

## 3. Results

### 3.1. Fused Deposition Modeling Optimization

The extrusion temperature for FDM was tested in the range from 225 °C to 240 °C in 5 °C increments. At 225 °C, the filament did not have sufficient adhesion to the base plane of the printer, while at 240 °C, the filament extruded out of the nozzle was difficult to control, as it was not viscous enough. The optimal extrusion temperature was found to be in the range between 230 °C and 235 °C.

**Figure 2:**
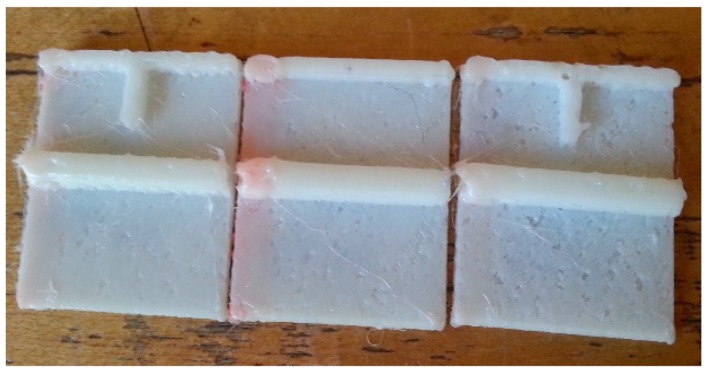
Test samples produced with fused deposition modeling (FDM) using travel speeds of the extruder (left to right) of 15, 20, and 30 mm/s.

The travel speed of the printer head significantly influenced the quality of the final build. Figure 2 presents the test samples produced with the optimized extrusion temperature, and with travel speeds of 15, 20, and 30 mm/s (left to right). The test samples had a geometry of 2.5 cm × 2.5 cm square, with a thickness of 1.5 mm. On top of the sample, there were two structures: a T-shaped wall placed at the edge of the test sample, and a microfluidic channel in the middle of the test sample. The microfluidic channel had an inner diameter of 1.5 mm and an outer diameter of 3 mm. As Figure 2 indicates, the best quality of the printed structures resulted with the extruder’s travel speed of 15 mm/s.

### 3.2. Stereolithographic Fabrication Optimization

**Figure 3:**
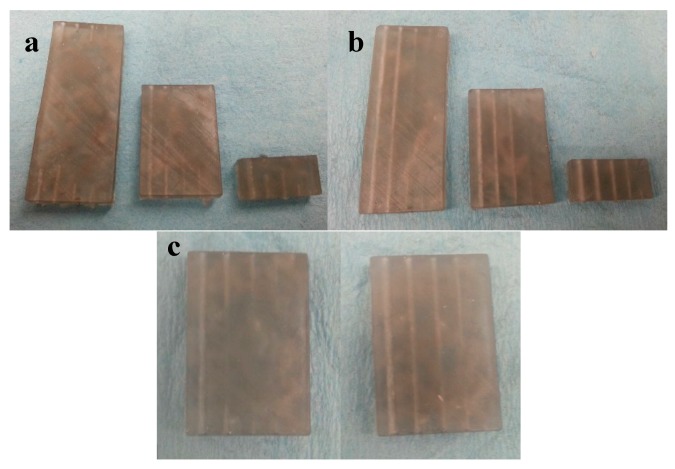
Test Samples containing microchannels with diameters of 2.1 mm, 1.7 mm, 1.4 m, 1.0 mm, and 0.7 mm (right to left). The test chip lengths are 5 cm, 3 cm, and 1 cm. Prolonging the isopropyl alcohol (IPA) soak from 30 min (**a**) to 6 h (**b**) helps with clearing the microchannels from uncrosslinked resin. (**c**) A comparison between IPA soaking cleaning (left) and IPA injection cleaning (right) for the 3 cm long microchannels. IPA injection cleaning achieved superior results in cleaning the channels from uncrosslinked resin.

For microfluidic parts produced with SLA, one of the major challenges was the process of clearing out the uncrosslinked resin from the enclosed microfluidic channel [67]. We evaluated the smallest channel diameter achievable with SLA by printing chips with 1 cm, 3 cm, and 5 cm long microchannels. Each sample contained five channels with different hydraulic diameters of 0.7 mm, 1.0 mm, 1.4 mm, 1.7 mm, and 2.1 mm (Figure 3a). The longer the channel and the smaller its diameter, the more difficult it was to clear the channel from uncross-linked resin. For example, all of the channels in the 5 cm long test chip were clogged, even with 30 min of soaking time in isopropyl alcohol (IPA).

**Figure 4:**
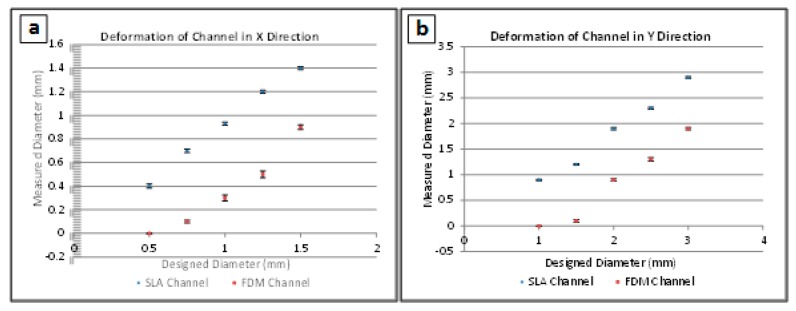
Deformations of hollow channels printed with Stereolithography (SLA) and Fused Deposition Modeling (FDM) technologies. A total of five samples were measured for each experimental point, and error bar heights represent one standard deviation. Designed vs measured channel diameter measurements are presented for (**a**) the deformation of channels in the X direction (width of the channels) and for (**b**) the deformation of channels in the Y direction (height of the channels).

**Figure 5:**
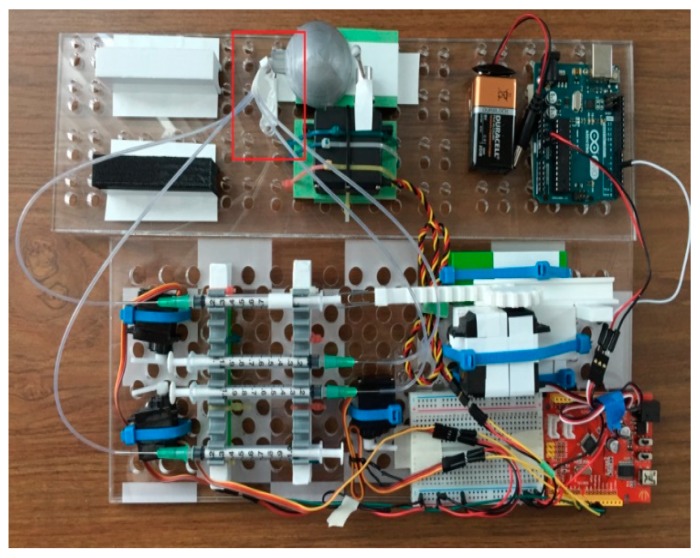
Top view of the assembled automated ELISA bioassay platform. Test wells are indicated by the red frame on the picture. See the caption of Figure 1 for a description of the individual components of the automated platform.

**Figure 6:**
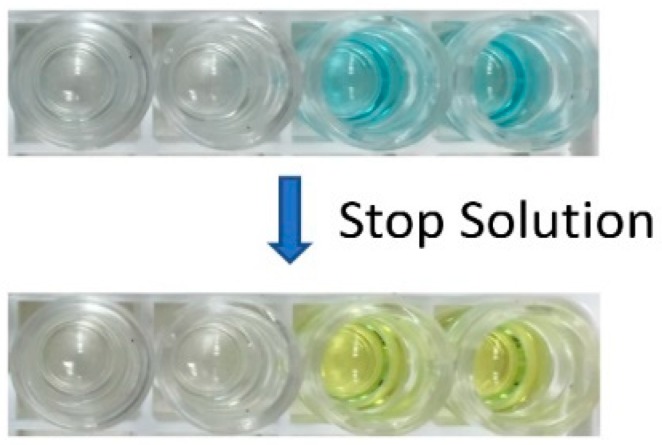
Colorimetric bioassay results after the stop solution is added. The wells with the blue solution are the positive controls and the colorless wells represent the negative controls.

Extending the IPA soak time of the SLA fabricated sample from 30 min (Figure 3a) to 6 h (Figure 3b) helped to clear out channels better. Further extending the soak time in IPA past 6 h did not result in any noticeable change, while the cross-linked resin started to deteriorate. The channels with diameters of 1.75 mm and larger were cleared out even in the 5 cm long test pieces.

For straight channels, it was possible to remove the uncross-linked resin by feeding a thin wire through the channel, but in case of tortuous channels (of sinusoidal or zig-zag geometry), this technique was difficult to execute successfully, as the wire could not navigate the bends of the tortuous channels. Instead, we implemented the manual injection of IPA with a syringe directly into the channels. This technique was more efficient and superior to the IPA soak method.

As can be seen on Figure 3c, the 3 cm long microchannels were still clogged after a 6 h IPA soak, while the IPA injection successfully cleared the microchannels in another sample of the same geometry. IPA injection is capable of clearing channels all the way down to microchannels with 1 mm diameter.

### 3.3. Channel Deformation

Channel cross-sections were distorted during FDM and SLA fabrication. Measurements in the X and Y directions (width and height of the channels’ cross-sections, respectively) were obtained for all channels and compared to the designed values. The cross-sections of all the channels were oval, meaning that the diameter in the Y direction was larger than in the X direction. Figure 4 compares the designed and measured diameters in the X and Y direction for channels made with both FDM and SLA.

It was seen that in all cases, SLA produced channel diameters that were more representative of the initial design, while FDM was far less accurate due to spreading of the liquid filament during printing. Both methods produced distorted channel heights due to the sagging of unsupported filaments. However, SLA-fabricated samples channels tended to sag less than the FDM-fabricated channels. The highest deviation observed corresponded to FDM-printed channels that were larger than 1 mm diameter in the X direction.

### 3.4. Performance of the Automated System

The finalized printed LOC platform was assembled using tubes to connect the syringes to the reservoir wells. The reagents contained within the syringes were injected into the wells (boxed in red in Figure 5). The dead volume of the system, comprising the inner space of the tubes connecting the syringes and the test well, equaled 0.14 ± 0.02 cm^3^ for each syringe. In order to compensate for that dead volume, correspondingly larger volumes were dispensed from the syringes.

An assay was performed using the automation process described in the Section 2.2 above. A close-up photo of the positive and negative results is provided in Figure 6. The wells with the blue solution were the positive controls, and the colorless wells represented the negative control. The positive control solution changed from blue to yellow when the stop solution was added, while the colorless negative controls did not change color. The positive and negative control color change was correct, signifying the successful demonstration of the automated malaria-Ab assay.

Five assays were performed on the automated platform. Each assay consisted of four test wells: two wells with positive controls and two wells with negative controls. The tests were performed using one well at a time. Subsequent test runs were performed by replacing the tubes and manually positioning them into another test well. All of the assays were performed successfully without any false readings. Minor test-to-test variations (within 0.05 cm^3^) of the dispensed wash did not affect the test results.

A Windows Nokia Lumia 521 (Nokia, Espoo, Finland) smart phone with the installed Color Catcher (Cloud Innovation Team, Austin, TX, USA) app was used to capture the RBG color code of the solution in the well at the end of each test. There was no need to withdraw the tubes from the test well during this image acquisition. While the color change was easily distinguishable with the naked eye, such captured color codes are useful for performing quantitative bioassays. The video of the operation of the automated bioassay platform during the test can be viewed online as part of the Supplemental Materials for this article.

## 4. Conclusions

We have reported on the fabrication of an automated colorimetric Malaria-Ab immunoassay platform based on readily-available parts, such as servomotors and disposable syringes. We have also outlined an alternative syringe-based automated assay that utilizes elastomeric reservoirs (printed using SLA) with programmable servomotors compressing elastomeric reservoirs to inject reagents or to wash a test well. The aspiration syringe actuated by a continuous servomotor facilitated the draining of test wells after specific assay steps.

To demonstrate the validity of our approach, we fabricated a hybrid system that included syringes for all of the reagents except for the wash, which was contained in an elastomeric dome reservoir. Successful validation of the automated platform was performed with positive and negative controls, and with the reagents included in the commercial malaria-Ab immunoassay kit.

The demonstrated syringe-based automation is easily expanded to a wide variety of assays to create flexible and affordable point-of-care platforms. Implementation of the platform is simplified by providing rural clinics with syringe kits with pre-measured reagent syringes sealed in metallized pouches. These pouches can be punctured by the syringe plunger during the corresponding assay step when conducting the bioassay. Additional validation is required before the application of the presented automated platform for qualitative (rather than quantitative) tests.

## Figures and Tables

**Table 1:** Malaria-Ab Enzyme-linked Immunosorbant Assay (ELISA) instructions as provided by the kit manufacturer and modified steps executed by the automated bioassay platform.

ELISA Step	Malaria-Ab ELISA Kit Instruction	Automated Assay Modifications
Sample	Add 50 μL of the undiluted sample to a coated well. Mix on a plate shaker for 30 s. Incubate (covered) at 37 °C for 30 min.	50 µL of sample is pipetted into the wells (O). Incubation occurs for 1 hr at room temperature.
Wash	Wash five times with 300 μL of working strength wash buffer. A short soak time of about 30 s is recommended between each wash cycle. Tap out excess liquid.	The well is flushed with 300 μL wash solution contained within the elastomeric dome (N) as the movable arm (M) compresses the dome. The arm is controlled by the servomotor (L) and Arduino board (J). The dirty wash solution is aspirated from the well using an aspiration syringe located on the bottom platform. The syringe is driven by 3D printed rack-and-gear set (B) and the servomotor (C). The wash/aspiration cycle is repeated three times.
Conjugate Incubation	Add 50 μL of diluted (1:10) conjugate to each well. Incubate (covered) at 37 °C for 30 min.	50 µL of diluted malaria conjugate is dispensed into the reagent well (O) from a syringe (F). The barrel of this syringe is pushed using a servomotor (H) controlled by the Arduino board (D). Incubation occurs for 30 min at room temperature.
Wash	See wash step above.	See wash step above.
Substrate Incubation	Add 50 μL substrate /chromogen Tetramethylbenzidine (TMB) mixture to each well. Incubate at room temperature for 30 min. As the substrate is photosensitive, it is recommended that the plate be protected from light during this incubation.	50 µL of TMB substrate solution is dispensed into the reagent well (O) from a syringe on the syringe platform (F). The barrel of this syringe is pushed using a servomotor (G) controlled by the Arduino board (D). Incubation occurs for 15 min.
Stop	Add 50 μL stop solution to each well. (Blue color changes to yellow).	50 µL of stop solution is dispensed into the reagent well (O) from a syringe (F). The barrel of this syringe is pushed using a servomotor (E) controlled by the Arduino board (D).
Detection	Read with fluorescent microplate reader at 450 nm (A450). Use of a reference filter at 620–690 nm will eliminate the effects of scratches, bubbles, etc.	A color change is accessed visually or via a cell phone positioned on the frame (P) with phone camera aligned to the reagent wells (O) to read the results using a colorimetric detection application.

## Supplementary Materials

The following are available online at http://www.mdpi.com/2072-666X/9/10/502/s1, Video S1: Validation of a syringe-based automated point-of-care malaria-Ab immunoassay.
